# Multi-contact functional electrical stimulation for hand opening: electrophysiologically driven identification of the optimal stimulation site

**DOI:** 10.1186/s12984-016-0129-6

**Published:** 2016-03-08

**Authors:** Cristiano De Marchis, Thiago Santos Monteiro, Cristina Simon-Martinez, Silvia Conforto, Alireza Gharabaghi

**Affiliations:** Division of Functional and Restorative Neurosurgery, Department of Neurosurgery, Eberhard Karls University, Otfried-Mueller-Str.45, 72076 Tübingen, Germany; Neuroprosthetics Research, Centre for Integrative Neuroscience, Eberhard Karls University, Tübingen, Germany; Laboratory of Bioengineering BioLab3, Department of Engineering, University Roma TRE, Via Vito Volterra 62, 00146 Rome, Italy

**Keywords:** Neuromuscular electrical stimulation, Multi-contact stimulation, EMG, M-wave, Hand function, Neurorehabilitation

## Abstract

**Background:**

Functional Electrical Stimulation (FES) is increasingly applied in neurorehabilitation. Particularly, the use of electrode arrays may allow for selective muscle recruitment. However, detecting the best electrode configuration constitutes still a challenge.

**Methods:**

A multi-contact set-up with thirty electrodes was applied for combined FES and electromyography (EMG) recording of the forearm. A search procedure scanned all electrode configurations by applying single, sub-threshold stimulation pulses while recording M-waves of the extensor digitorum communis (EDC), extensor carpi radialis (ECR) and extensor carpi ulnaris (ECU) muscles. The electrode contacts with the best electrophysiological response were then selected for stimulation with FES bursts while capturing finger/wrist extension and radial/ulnar deviation with a kinematic glove.

**Results:**

The stimulation electrodes chosen on the basis of M-waves of the EDC/ECR/ECU muscles were able to effectively elicit the respective finger/wrist movements for the targeted extension and/or deviation with high specificity in two different hand postures.

**Conclusions:**

A subset of functionally relevant stimulation electrodes could be selected fast, automatic and non-painful from a multi-contact array on the basis of muscle responses to subthreshold stimulation pulses. The selectivity of muscle recruitment predicted the kinematic pattern. This electrophysiologically driven approach would thus allow for an operator-independent positioning of the electrode array in neurorehabilitation.

## Background

Functional Electrical Stimulation (FES) is a widely used technique for inducing muscle contraction. FES induces muscle activation through the application of currents that are able to excite the axons of the motor-neurons innervating the target muscles. This technique has been extensively studied for both training and rehabilitation purposes. A number of studies have shown the effectiveness of FES for improving muscle function in different central nervous system (CNS) disorders such as stroke [1], tremor [2], multiple sclerosis [3, 4] or spinal cord injury [5] for both the upper and lower extremity [6].

Particularly, the hand function is a major target of FES in CNS disorders due to its relevance for quality of life [7] and activities of daily living [8] in both the acute and chronic phase [9]. In this context, FES might enhance hand functionality when physical therapy alone is ineffective [10–14].

Due to its simplicity, most FES applications use pairs of electrodes in bi- or monopolar configuration [15]. Depending on the shape, size and positioning of the active and return electrodes, different current paths are induced in the motor axons underlying the stimulating electrodes, and different muscles are thus targeted. In particular, the larger the electrode, the less specific the stimulation becomes [16]. When aiming at the recovery of specific hand functions, it is though essential to induce specific muscle contraction. However, surface electrode stimulation is generally limited by its lack of selectivity. This is particularly critical when targeting specific hand movements due to the complexity of the muscle topography within the forearm. A more selective stimulation can be achieved with the use of an electrode array [17, 18], which needs, however, to be adjusted in terms of contact selection for each individual subject [19]. Such an approach would facilitate the stimulation of specific muscles for selective movements, physiological synergies or more complex patterns relevant for activities of daily living.

Up till now, different procedures have been proposed to detect the optimal FES site [20–25]. All of these methods have in common that they probe stimulation induced muscle twitch responses, e.g. by capturing the kinematic response of the targeted degrees of freedom. However, this kinematic response might be diminished or even absent e.g. in stroke patients due to spasticity. Moreover, the application of high current levels could lead to pain and muscle fatigue if applied repeatedly. However, electrical stimulation pulses evoke an electrophysiological response as well, i.e. the M-wave, which closely relates to muscle fiber recruitment. This electrophysiological response is detectable even when sub-threshold stimulation pulses are applied, which do not elicit kinematic responses, i.e. movements. The M-wave represents the synchronous muscle response to the action potentials traveling ortho-dromically through the axon of the motor neuron; this signal can be detected quickly and reliably by using standard electromyographic (EMG) measurements over the target muscle, thereby providing an objective and specific measure of the neuromuscular effects of electrical stimulation [26–28].

The present study is based on the hypothesis that the electrophysiological response to sub-motor threshold single pulse neuromuscular electrical stimulation will predict the effects of supra-motor threshold FES bursts. The mapping procedure is based on M-wave recordings from multiple muscles of the forearm during single pulse stimulation. The functional relevance of the selected stimulation sites is verified after the mapping with stimulation bursts producing specific movements of fingers and wrist extension/deviation. This electrophysiologically driven approach is expected to lead to automatic identification of selective electrode configurations of a multi-contact array for functional hand opening. The feasibility of selective muscle recruitment and kinematic pattern prediction is tested here in healthy subjects, while the ultimate goal will be to translate this technique to neuro-rehabilitation for the recovery of hand function in stroke patients.

## Methods

This section is divided into three main sub-sections. The first part describes the setup used for applying electrical stimulation, recording the EMG responses and measuring the kinematic output. The second part describes all the procedures adopted during the experimental sessions, including the stimulation protocol and data processing. The third part describes the offline analysis procedures for performance evaluation.

### Participants

Eight young healthy subjects (27.4 ± 2.6 years old) participated in the study after providing written informed consent. The study was carried out according to the principles of the declaration of Helsinki and was approved by the ethics committee of the medical faculty of the University of Tuebingen. All subjects were right handed, and the stimulated forearm was the left one.

### Experimental setup

#### Neuromuscular electrical stimulation and EMG recording

**Fig. 1:**
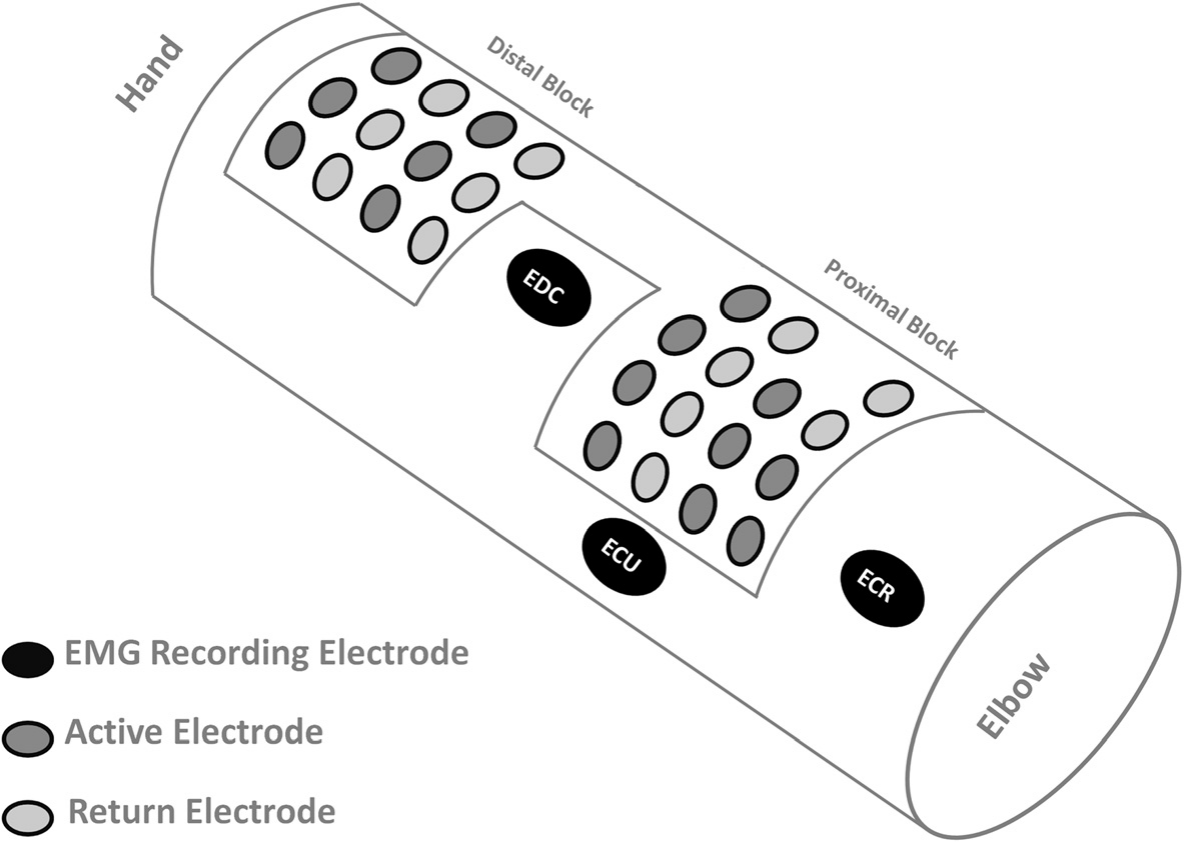
Multi-contact set-up visualized for the left arm: Electrode array for FES with a fixed configuration of two blocks, i.e. a proximal and a distal (close to the hand) block with 15 and 12 contacts respectively, and 3 flexible electrodes for EMG recording

FES was provided through a surface electrode array (RehaStim2+, HASOMED GmbH, Magdeburg, Germany). The array was composed of two main blocks with fixed electrode positions: a proximal block containing 15 electrodes with 9 stimulation and 6 return contacts, and a distal block containing 6 stimulation and 6 return contacts arranged in a 3×4 configuration, as shown in Fig. 1. The stimulation/return electrodes were oval shaped (gold, 1,4 cm x 0,8 cm) and had an inter-electrode, center-to-center distance of 2 cm and 2,2 cm in the vertical and horizontal direction, respectively. The contact quality was improved through the use of a conductive gel sheet placed on each electrode. A stimulation configuration was defined as a pair composed of an active and a return electrode and will be referred to as *stimulation pattern*. Based on this array configuration, 180 different FES stimulation patterns could be studied.

EMG signals were recorded with a monopolar configuration;, the recording electrodes (Ag/AgCl, AMBU Neuroline) were positioned over the three targeted forearm muscles, i.e. *Extensor Digitorum Communis* (EDC), *Extensor Carpi Radialis* (ECR) and *Extensor Carpi Ulnaris* (ECU), while reference and ground electrodes were positioned over the olecranon. EMG electrodes were positioned based on anatomical landmarks identified in previous studies aiming at recording selective EMG activity from forearm muscles [29, 30]. The correct positioning was supported by palpation during targeted movements [29]. EMG signals were sampled at 5000 Hz (BrainAmp DC amplifier; Brain Products GmbH, Gilching, Germany) and digitized through a 16 bit A/D converter.

#### Kinematics recording

Finger and wrist movements were recorded by using a DG5 VHand 3.0 Glove device (DGTech Engineering Solutions, Italy, http://www.dg-tech.it/vhand3) providing a resolution of 12 bit for the bending sensors and of 0.01°, 0.01° and 0.05° for pitch, roll and yaw, respectively, for the attitude estimation of the hand. This devise was equipped with five resistive bending sensors that provided a measure of bending for the five fingers. A tri-axial Inertial Measurement Unit placed on the dorsal side of the hand provided the pitch, yaw and roll angles, respectively, corresponding to finger and wrist flexion/extension, radial/ulnar deviation and forearm pronation/supination angle. Kinematic data were recorded with a sampling frequency of 100 Hz and digitized with 16 bits.

### Experimental protocol

During the experimental sessions, the subjects sat comfortably in a chair with the forearm kept in a relaxed pronated position, while the wrist was kept relaxed and free of any constraints. Before the stimulation part of the experimental protocol, the subjects were asked to perform 10 natural hand openings. This template of hand openings was used as a reference for both the evaluation of the behavioral outcome of FES and for post-hoc data analysis.

The experimental protocol consisted of two main parts that will be described in two separate sections. The first part describes the single pulse scanning procedure: all possible stimulation patterns were used to identify subsets of optimal stimulation patterns for each of the three analyzed muscles. M-waves generated by the application of each pulse were then analyzed. The second part describes the application of FES bursts at the previously identified stimulation patterns with the aim of eliciting targeted movements of the wrist and fingers. The setup, including glove and electrodes donning, scanning the array and EMG offline analysis, took approximately 15 min.

#### Scanning the array and selecting the optimal stimulation patterns

In order to determine subsets of optimal stimulation patterns, the electrode array was scanned with single stimulation pulses. Each pulse was biphasic and applied with a fixed total pulse width of 500 μs. This pulse-width value was chosen after a pilot study in order to reduce inter-subject variability of the threshold and stabilize the current values [18]. First, single pulses were delivered at 10 mA at each stimulation pattern. The subjects were asked to report any uncomfortable sensation. The contacts inducing any kind of discomfort were excluded. The remaining patterns were then stimulated at three different amplitudes (9 mA, 10 mA and 11 mA) with each current level applied three times at each pattern, i.e. resulting in nine pulses per pattern. These intensities were below the kinematic threshold when applied as single pulses, i.e. they did not elicit functional finger extension, wrist extension and ulnar deviation. Single pulses were applied every 300 ms in order to avoid summation effects and muscle contraction by allowing a complete muscle relaxation before the application of the next pulse [31]. This scanning procedure led to the application of a maximum of 1620 single pulses and was completed in approximately 8 min.

After the completion of the scanning procedure, a EMG offline analysis was performed with a custom written MATLAB script (The MathWorks, Natick, MA). After visual inspection of the data quality, EMG signals were band pass filtered (10Hz-1000Hz, 3rd order Butterworth filter) in order to remove low frequency movement artifacts and high frequency noise. When present, power line noise interference and the first two higher order harmonics were removed with a 5th order Butterworth band stop filter around 50Hz. In order to eliminate the stimulation artifact from the analyzed EMG channels, a threshold algorithm was applied to the absolute value of the EMG signal derivative, and the signal was blanked within the detected spike intervals. This procedure led to EMG signals containing only the muscular response to the applied stimulation. In order to compare the responses across different targeted muscles, each EMG signal was normalized with respect to the amplitude of the maximum absolute value of the processed EMG, corresponding to the maximum evoked M-wave among all the provided pulses and stimulation patterns.

**Fig. 2:**
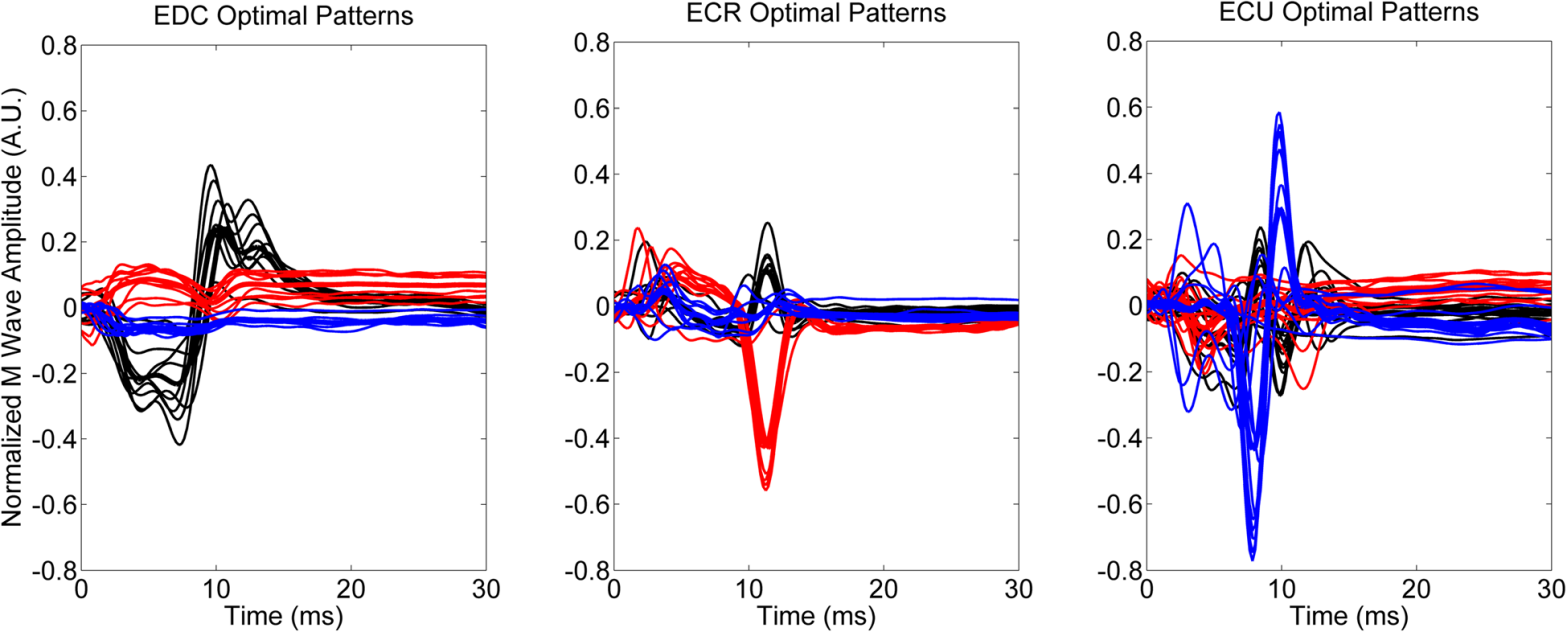
M-waves during the scanning procedure for the identified optimal stimulation patterns for one representative subject (black: EDC, red: ECR, blue: ECU). Thick lines represent the average M-wave for the corresponding set of stimulation patterns

After EMG pre-processing, the muscular response to each delivered pulse for each muscle was analyzed in a time window from 2 ms to 40 ms after the identified stimulation artifacts. The muscular response was quantified in terms of features extracted from the recorded M-waves. The peak-to-peak amplitude was calculated for the M-waves recorded from each muscle. The selection of the optimal stimulation patterns was based on the selectivity of the recorded M-waves: for each pattern, the median M-wave was calculated from the muscle response of the nine applied pulses. A stimulation pattern was considered optimal for a muscle if the peak-to-peak of the median M-wave was at least twice as a high as the median M-wave recorded from each of the other two muscles (Fig. 2).

#### Stimulating at the optimal patterns

After identifying the subset of optimal stimulation patterns for each muscle, each pattern was stimulated individually through the application of a 5 s FES burst. Each burst was applied with a frequency of 30 Hz and a pulse width of 500 μs. All identified patterns were first stimulated with the current level used during the test procedure (10 mA as default value). If no movement was present at the baseline level, the amplitude was gradually increased in steps of 2 mA until the kinematic threshold was reached and a visible movement elicited. The stimulation of the optimal EDC pattern was carried out in two different starting positions of the forearm: a reference position of the arm, identical to the one during the scanning procedure, and a 90° neutral forearm position. This procedure was carried out to study the robustness of the obtained patterns to a rotation of the forearm, during which the relative rotation of the skin on the muscles might lead to muscles sliding with respect to the original electrodes position. For both conditions, the subject was asked to close the hand – without providing resistance to the elicited movement – before the application of each FES burst. For ECR (wrist extension) and ECU (wrist ulnar deviation) the same procedure was applied, but using only the reference forearm posture; this additional part of the experimental protocol was performed to check the sensitivity of the stimulation patterns to generic arm movements.

### Data analysis

Percentage of fingers extensions with respect to template opening was used as a measure of behavioral outcome for finger extension, as indicated in the following equation:

1 $$ Fex{t}_i=100\frac{F{E}_i}{T_{FEi}} $$

Where *Fext*_*i*_ denotes the metric for the extension of the *i-th* finger, *FE*_*i*_ denotes the value measured by the glove for the extension of the *i-th* finger, and *T*_*FEi*_ indicates the value measured during template hand opening for the same finger.

For the other degrees of freedom (i.e. wrist extension and ulnar deviation) absolute angles were used as measures of behavioral outcome. In order to assess the performance of the stimulated patterns, a measure of selectivity was introduced for each stimulated pattern targeting a specific degree of freedom (fingers extensions, wrist extension and ulnar deviation). Assuming that the three targeted degrees of freedom are independent, we can represent each of them as one of three orthogonal directions in a Cartesian reference system and define a measure of selectivity related to the cosine of the elevation angle in spherical coordinates as follows:

2 $$ S= \cos \left(\theta \right)=\frac{A}{\sqrt{A^2+{B}^2+{C}^2}} $$

Where A is the targeted degree of freedom, B and C are the non-targeted degrees of freedom and θ is the angle between the desired direction and the obtained one. The parameter S provides a direct measure of selectivity for each stimulated pattern with respect to the targeted degree of freedom, assuming values in the range [0:1].

In order to evaluate the effectiveness of the proposed method, we compared the behavioral outcomes emerging from the stimulation of the optimal patterns for the targeted degree of freedom A_OPT_ with a set of control measurements, defined as the behavioral outcome A_CONTROL_ when the optimal patterns for B and C were applied. This procedure was adopted because it was not possible to apply effective control measurements deriving from random patterns, due to fatigue and potential pain. In order to assess the statistical significance of the difference, we performed multiple Wilcoxon tests. The difference was considered statistically significant for values of *p* < 0.05.

## Results

We explored 1440 stimulation patterns during the scanning procedure, i.e. 180 patterns in each of the 8 subjects; 50 stimulation patterns (about 3 %) were excluded from further evaluation due to discomfort. In 12 cases, the selected pattern, which was comfortable during the scanning procedure, revealed discomfort, when stimulated with 30Hz bursts at baseline intensity (5 cases for EDC, 2 cases for ECR, 5 cases for ECU). For these patterns the intensity was not increased and they were excluded from further analysis.

The presented approach was successful in detecting the stimulation patterns which were effective for functional hand opening in all subjects. This allowed reducing the total number of possible stimulation patterns (*n* = 180) to the sufficient ones for hand opening (meanFE = 12.5, minFE = 5, maxFE = 29), wrist extension (meanWE = 7.4, minWE = 6, maxWE = 10) and ulnar deviation (meanUD = 8.7, minUD = 3, maxUD = 15), respectively.

With regard to hand opening, the targeted template hand opening with a complete extension of all fingers could be achieved with 66 % of the selected stimulation patterns; in 21 % of the patterns, an incomplete extension of the fingers was achieved or additional wrist deviation occurred. In only 13 % of the patterns, M-waves did not predict the targeted movements.

To better assess the efficacy of stimulation for functional hand opening, the findings were divided into two classes on the basis of the functional relevance of the finger extension (i.e. functional or non-functional extension). The finger extension was defined as functionally relevant when corresponding to two thirds of the full extension from a closed finger position. The number of functionally relevant stimulation patterns was highly dependent on the subject, ranging from a minimum of 2 to a maximum of 21 across subjects (mean number of functional patterns = 8.9). In general, the functional finger extension was closely mimicking the template hand opening.

For the different muscles, a different number (N: mean/median) of 2 mA intensity increments from the baseline value were necessary to achieve the results presented in this section: N_EDC_ = 2.25/2, N_ECR_ = 2.71/3, N_ECU_ = 1.25/1. ECU required generally lower currents for eliciting movements, because in the reference position ulnar deviation was the only degree of freedom that had not to overcome gravity.

### Optimal patterns *vs* control measurements

**Fig. 3:**
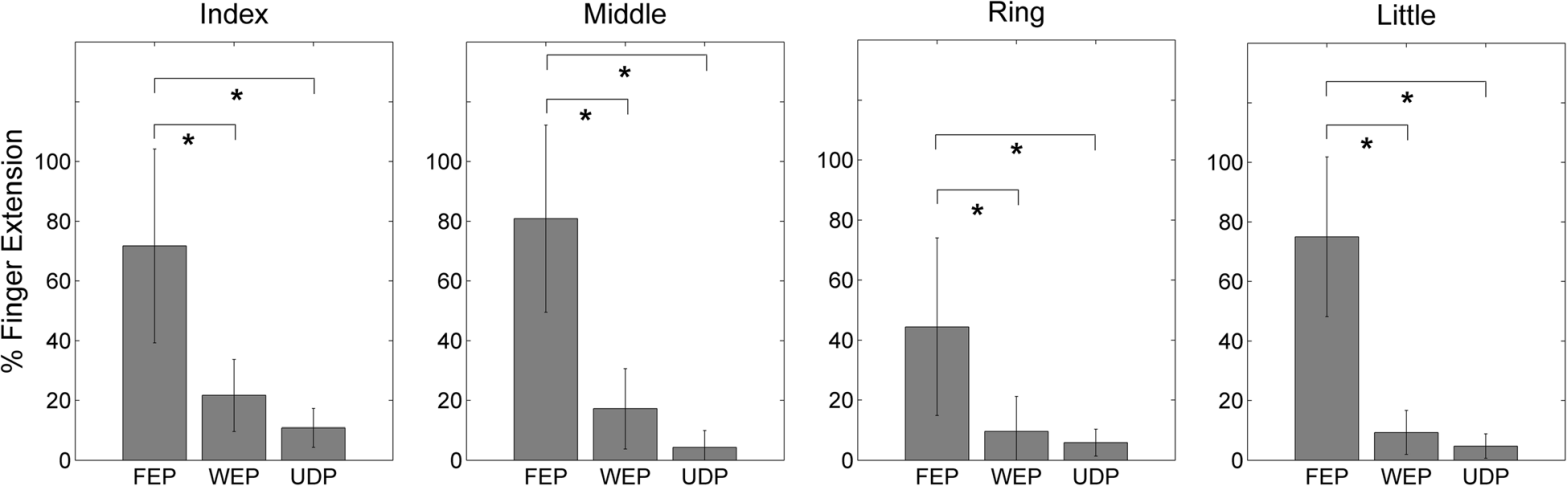
Finger extension movements (median value ± median absolute deviation) comparing different stimulation patterns (FEP = Finger Extension Pattern, WEP = Wrist Extension Pattern, UDP = Ulnar Deviation Pattern). In this case WEP and UDP constitute the control measurements for FEP

**Fig. 4:**
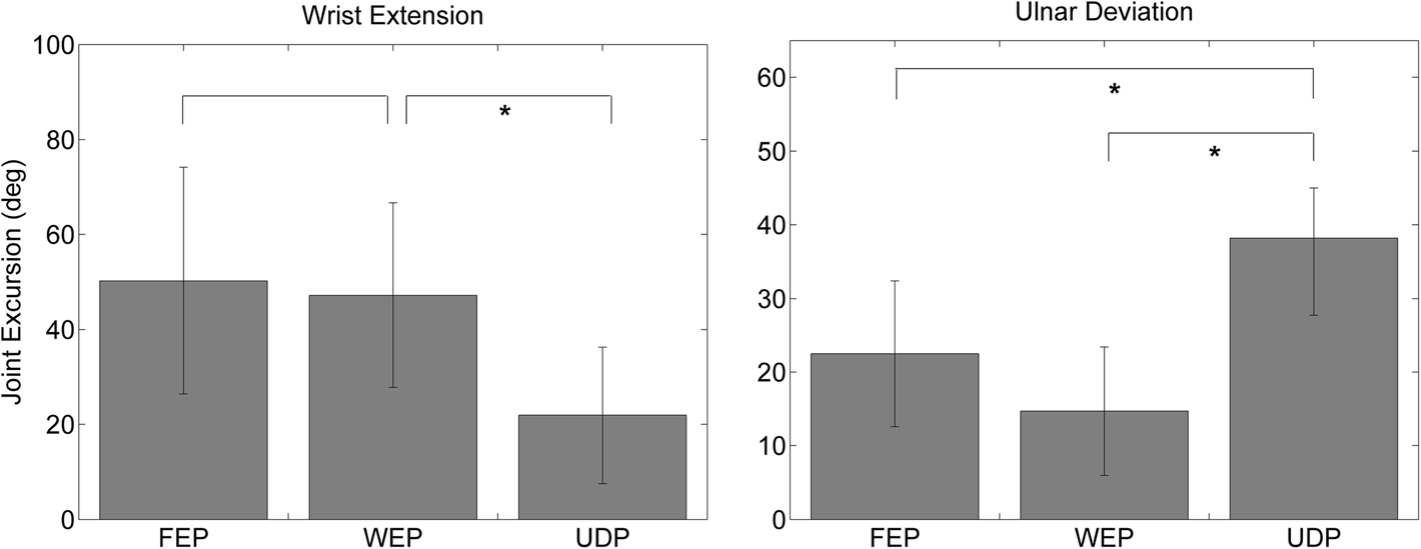
Wrist extension and ulnar deviation movements (median value ± median absolute deviation) comparing different stimulation patterns (FEP = Finger Extension Pattern, WEP = Wrist Extension Patterns UDP = Ulnar Deviation Patterns). Left panel: FEP and UDP constitute the control measurements for WEP. Right panel: FEP and WEP constitute the control measurement for UDP

The behavioral outcome obtained with stimulation at the optimal pattern was significantly higher than that obtained from the control measurements. More specifically, with regard to hand opening, the extension of all fingers was significantly higher with the optimal Finger Extension Patterns (FEP) as compared to the corresponding control measurements (Wrist Extension Patterns, WEP, and Ulnar Deviation Patterns, UDP) (Fig. 3). With regard to wrist extension, the WEP was significantly more effective that UDP, but not than FEP (Fig. 4, left). Moreover, ulnar deviation was significantly more effective during UDP as compared to WEP and FEP (Fig. 4 right).

### Selectivity with optimal stimulation pattern

**Fig. 5:**
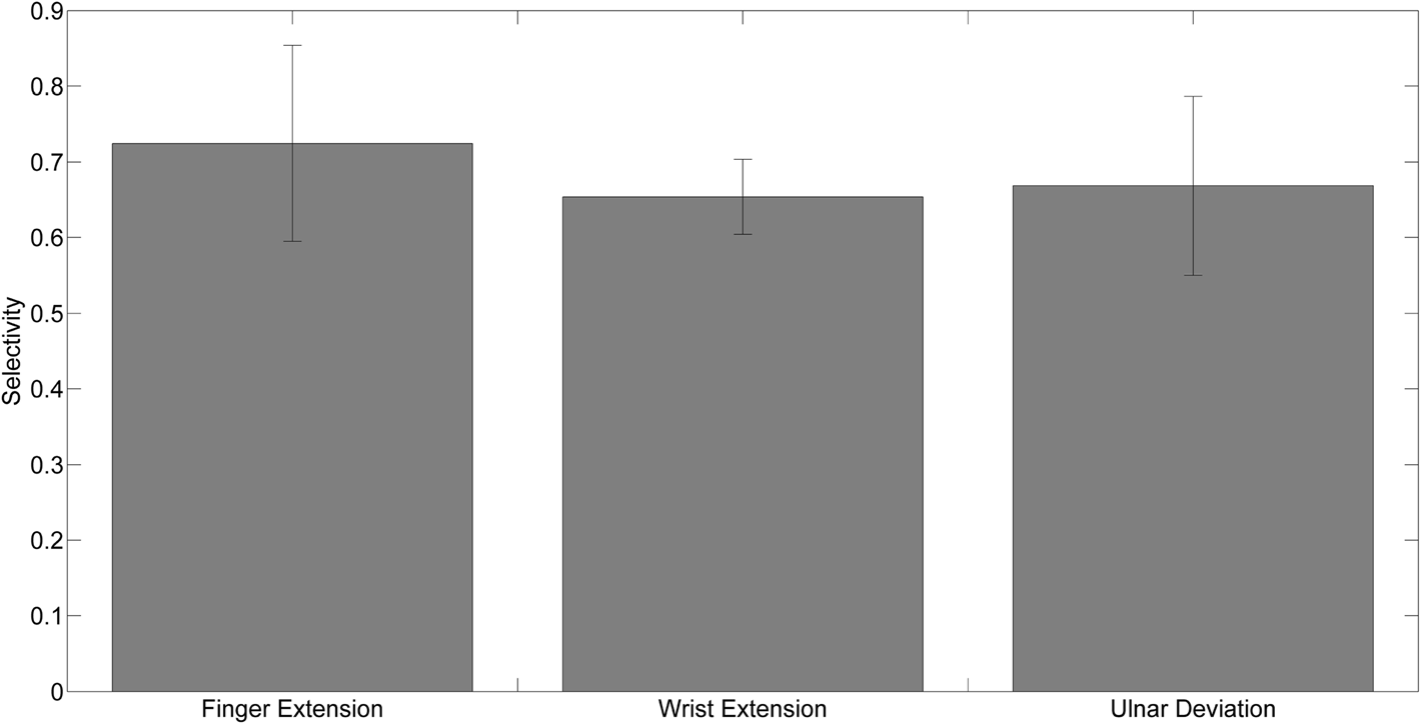
Measure of selectivity under the optimal stimulation pattern for finger extension, wrist extension and ulnar deviation, respectively. A high selectivity level was obtained for each of the identified sets of optimal patterns

All movements obtained by the respective optimal stimulation pattern (FEP for hand opening/finger extension, WEP for wrist extension and UDP for ulnar deviation) proved to be highly selective. When the parameter S was higher than 0.6, this indicated that the contribution of the non-targeted movements was negligible in comparison to the targeted ones (Fig. 5).

### Hand opening with a rotated forearm position

**Fig. 6:**
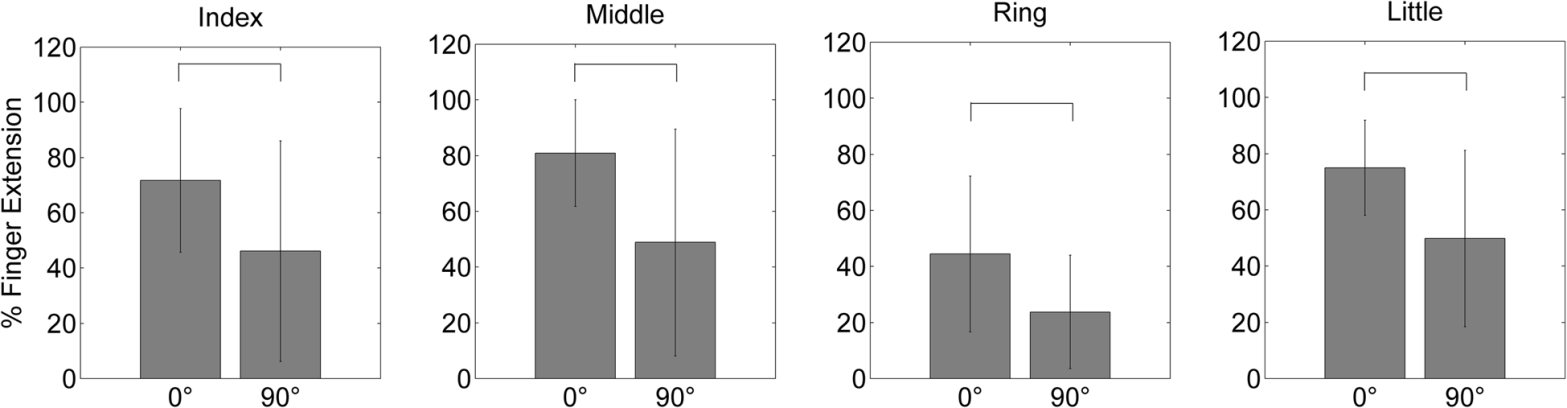
Finger extension (median value ± median absolute deviation) in the reference position (0°) and in the rotated forearm position (90°): No differences were found for each of the fingers. The amount of hand opening was thus comparable for the two forearm postures

When applying the identified optimal FEP during changed forearm/hand position no differences were found with regard to the individual finger extension. The identified patterns were thus independent of the forearm pronation/supination postures, i.e. with a 90° rotation of the forearm (Fig. 6).

## Discussion

We introduced a novel FES mapping procedure with a multi-contact array. This approach allowed identifying a subset of electrodes that were optimal for eliciting functional hand opening with selective muscle recruitment for achieving the targeted movement. Furthermore, other movements, such as wrist extension and ulnar deviation, could be elicited as well with detecting a different optimal electrode configuration for selective muscle recruitment. Thereby, the presented procedure facilitated the detection of both muscle specific and behaviorally relevant electrode configurations.

### Innovation of the study

With respect to previous studies aiming at identifying the optimal stimulation patterns for eliciting selective hand and wrist movements, this is the first to fully rely on an electrophysiologically-based approach. Moreover, this approach is novel in using a variable location for both the active and return electrodes; the use of a set of different return electrodes on the array led to an increased amount of available stimulation patterns. This aspect might play an important role when aiming at an operator-independent FES positioning [17, 32]. Once the optimal muscle-selective stimulation patterns have been identified, further computational methods could be applied to generate even more specific movement patterns [24, 33].

Analyzing the muscle response to specific stimulation patterns through the use of surface EMG allows the extraction of specific features from the signal. In this study we studied the main muscles controlling the two kinematic degrees of freedom of the wrist (flexion/extension and radial/ulnar deviation) and the metacarpal joints of the fingers. However, increasing the sources of EMG recording even further would allow a selective identification of optimal patterns for a higher number muscles and of degrees of freedom: the thumb movement, for example, was not explicitly targeted in this study; however recording EMG from the M. abductor pollicis brevis could probably lead to a selective control of the thumb aperture as well allowing an independent control of the thumb.

Another advantage of the present approach is that it allows detecting the optimal stimulation pattern with relatively low current intensities. All previous methods necessitated supra-threshold stimulation, both in single pulse approaches [22] and in FES burst applications [20], since they used kinematics as the primary read-out. The approach proposed in this study overcomes potential limitations of applying supra-threshold stimulation, i.e. the risk of fatigue and pain, and could constitute a solution for e.g. stroke patients with a disturbed kinematic response due to spasticity.

#### Potential Application for Neurorehabilitation

The selective activation of a single muscle, as shown in this study, or of a specific group of muscles, might be of particular relevance for neurorehabilitation where particularly the interplay between agonist and antagonist muscles might be disturbed. After stroke for example, the neuro-muscular system might be impaired to a degree that the resulting spasticity would limit the detection of any muscle twitches. In this view, the proposed method might be particularly useful for those severely affected patients who show no residual hand movement at all. This technique might moreover allow a fine tuning of stimulation parameters as well, e.g. by disentangling the involvement of central pathways through spinal reflexes [26, 34, 35] and the peripheral contribution of the stimulation as detected with the M-wave, thus potentially limiting the stimulation induced muscle fatigue [36].

Furthermore, the effectiveness of FES interventions may depend on the targeted function and the stimulation specificity. From a motor learning perspective, the ability to select a single hand function could potentially enhance motor recovery and facilitate CNS plasticity [37]. Especially, after neurological injuries such as stroke, and particularly in those therapeutic scenarios seeking to close the loop between motor intention/attempt and assistive technology, a targeted activation of the periphery might be essential [38, 39]. However, further research is necessary along these lines to better understand the physiological effects of FES on the CNS, and particularly on cortical connectivity [40]. With regard to the cortical physiology during peripheral input [41, 42], recent findings in the field of brain-robot interfaces may inform FES applications as well [43], with the goal of bridging the abilities and cortical networks of motor imagery and motor execution [44–46] and strengthening corticospinal connectivity [47].

#### Possible limitations and future perspectives

Peak-to-peak values of M-waves were sufficient to detect optimal patterns, even though this approach did not always result in complete finger extensions. There was also no significant correlation between the M-wave peak-to-peak value and the quality, i.e. functional characteristics, of the finger extension. A more careful inspection of the recorded signals may help to further differentiate between functional and non-functional extensions. This could possibly be achieved by extracting additional features from the recorded M-wave besides the peak-to-peak values used in this study, e.g. power and area of the M-wave.

Due to space limitations for positioning the EMG electrodes on the dorsal side of the forearm, we used monopolar instead of bipolar recordings. Although this approach might be less selective, noisier, and more affected by cross-talk during simultaneous recording and stimulation, it nevertheless resulted in the identification of a relevant number of selective patterns. However, we might have missed some stimulation patterns due cross-talk among EMG channels. Future studies need to explore whether bipolar EMG recordings might improve the scanning procedure and lead to a higher number of selective stimulation patterns.

In neurological patients with central nervous system damage, e.g. stroke, the peripheral pathways are intact [31] and M-waves can be elicited similarly to the findings in healthy subjects studied here. For practical application, however, EMG signal quality needs to be carefully considered for the effective application of the proposed technique, due to potential atrophy [48, 49]. In this context, electrode arrays allowing concurrent recording and stimulation with a higher spatial specificity, i.e. lower inter-electrode distance, could potentially improve the selectivity of the proposed approach. Future studies could simultaneously record and stimulate both sides of the forearm, integrating the selective activation of flexor muscles in a bid to develop a system that is able to elicit selective hand opening and grasping together with the functional stabilization of the wrist.

## Conclusions

This study has introduced a novel method to automatically select stimulation electrodes for FES. The protocol identifies a small subset of electrodes from an array entirely based on the electrophysiological response to stimulation. This technique is capable of eliciting a full hand opening. The complete procedure is fast, comfortable and precise with regard to the targeted muscles. This could be particularly important for rehabilitation protocols addressing muscle synergies and activities of daily living.
